# Influence of Polypropylene, Glass and Steel Fiber on the Thermal Properties of Concrete

**DOI:** 10.3390/ma14081888

**Published:** 2021-04-10

**Authors:** Marcin Małek, Mateusz Jackowski, Waldemar Łasica, Marta Kadela

**Affiliations:** 1 Faculty of Civil Engineering and Geodesy, Military University of Technology, ul. Gen. Sylwestra Kaliskiego 2, 00-908 Warsaw, Poland; marcin.malek@wat.edu.pl (M.M.); waldemar.lasica@wat.edu.pl (W.Ł.); 2 Building Research Institute (ITB), ul. Filtrowa 1, 00-611 Warsaw, Poland; m.kadela@itb.pl

**Keywords:** building sector environmental impact, waste and byproduct reuse, fiber-reinforced concrete, thermal properties, material properties

## Abstract

The variety of approaches to tackle climate change reflects the size of this global problem. No technology will act as a panacea to cure the greenhouse gas emissions problem, but new building materials with byproducts or even wastes have the potential to play a major role in reducing the environmental impacts of the building sector. In this study, three potential solutions of concrete with dispersed reinforcement in the form of recycled fibers (polypropylene, glass and steel) were examined. The aim is to present a detailed analysis of the thermal properties of new building materials in an experimental approach. Concrete mixtures were prepared according to a new, laboratory-calculated recipe containing granite aggregate, a polycarboxylate-based deflocculant, Portland cement (52.5 MPa) and fibers. This experimental work involved three different contents of each fiber (0.5%, 0.75% and 1.0 wt.%), and all tests were carried after the complete curing cycle of concrete (28 days).

## 1. Introduction

The topic of industrial development is being more and more often taken up together with topics of sustainable development and a circular economy as indisputably related. These discussions have led to the creation of many projects aimed at providing environmentally friendly solutions that can be used in the industry [1,2,3,4,5]. One of the top projects in the building sector is Advancing Net Zero, which is the World Green Building Council’s global project working toward total sector decarbonization by 2050. Its main task is to develop tools, programs and resources to promote the urgency and achievability of net-zero carbon buildings and build industry capacity to deliver them. With the help of Green Building Councils (GBCs) across the network, they are trying to achieve two goals: (1) all new buildings must operate at net-zero carbon from 2030 and (2) 100% of buildings must operate at net-zero carbon by 2050 [6,7].

Responsibility for upward of 5% of anthropogenic greenhouse gas emissions is for the cement industry to bear as its main building material nowadays [8,9,10]. The largest source of carbon dioxide emissions (around 50%) is CO_2_, which comes during lime formation in the limestone heating and decarbonizing process, including all phases, namely raw material preheating, calcination, clinker burning and clinker cooling [11,12,13]. As the cement production process is highly energy demanding, a large amount of fuel is burned irreversibly. Fuel emissions account for 40% of total CO_2_ from the cement manufacturing process. Therefore, both cement substitutes [14,15,16,17] and alternative sources of energy [18,19,20,21] that can be used in concrete and cement production are sought, as presented in the current research. Furthermore, the building sector is consuming huge amounts of energy, as existing buildings need electricity and heating and cooling systems [22,23,24,25]. In China only, energy use for buildings is responsible for 5% of global energy-related emissions [26]. Thus, it is necessary to seek environmentally friendly solutions able to reduce the negative impact on the environment by the building sector. In this study, new construction material is proposed as a partial solution that may contribute to reducing environmental damage caused by the construction industry, as it contains fibers derived from wastes. A similar approach can also be observed in recent studies, as authors also tried to use waste as dispersed reinforcement in composites [27,28,29]. 

The thermal properties of materials have been studied widely in recent years [30,31,32,33]. Different materials react to the application of heat differently, but their response can be measured according to the functional characteristics of temperature or heat [34]. The thermal behavior of solids, i.e., the response of solid material to thermal change, often refers to three main properties, such as thermal conductivity, thermal diffusivity and specific heat [35,36,37]. Even a small decrease in thermal conductivity indicates lower heat transfer [38], which, in this case, is highly requested and can lower energy use for heating and cooling. Therefore, many studies focus on the modification of concrete mixtures using a byproduct [39,40] or waste material [41] as its current main building material. A wide range of recycled fibers available on the market, e.g., steel, glass and polypropylene, can be used not only as distributed reinforcement [42,43] but also to change the thermal properties of concrete [44,45,46,47,48]. Most studies focus on achieving higher mechanical properties of hardened concrete, such as compressive strength, flexural strength and split tensile strength, as they are crucial for construction sustainability [49]. However, they should be tested together with thermal properties, which are responsible not only for the comfort of the user but also for heat loss and the subsequent overheating of buildings. 

The thermal properties of fiber-reinforced concrete have been studied by many authors in recent years [50,51,52]. Compared to unreinforced concrete, fiber-modified concrete has shown a significant change in thermal properties, as reported by Ozger et al. [48]. By replacing part of sand with nylon fibers (5.000 g/m^3^ amount), they obtained around a 16% increase in thermal conductivity and around a 50% increase in specific heat compared to plain concrete. A similar increase in values of fiber concrete thermal conductivity was also obtained by Zhang et al. [50]. The simple 0.8% addition of carbon and glass fiber 6 mm in length (with a constant 0.5 water/cement ratio) led to a 14.3% and 12.7% increase compared to plain concrete, respectively. However, the 2% addition of 6 mm polypropylene and 6 mm basalt fiber resulted in a thermal conductivity decrease (13.2% for polypropylene fiber and 7.8% for basalt fiber). Thus, the thermal properties are dependent on the type of fiber. Furthermore, Liu et al. [53] arrived at the same conclusion. Tested carbon and steel fiber concretes (1.5% addition) showed a 5.1% and 11.8% increase (with a 0.5 water/cement ratio) for thermal conductivity, respectively. The difference in results between Liu et al. [53] and Zhang et al. [50] is due to the dimension and disparity amount of carbon fiber. Another type of fiber, polyvinyl alcohol fiber, was tested by Li et al. [54]. The decreasing trend in the thermal conductivity and specific heat of concrete with the fiber addition ranging from 1.5% to 2.2% was determined. The thermal properties of cellular alkali-activated fly ash concrete with glass fiber addition were tested by Stolz et al. [55]. The high and constant quantity of glass fiber (26.8 kg/m^3^) allowed achieving thermal diffusivity in the range of 0.18–0.20 mm2/s, thermal conductivity in the range of 0.23–0.31 W/mK and specific heat in the range of 1.19–1.70 MJ/m3K, depending on the amount of sodium silicate, sodium hydroxide and fly ash used for concrete mixtures.

The main goal of this study is to examine the effect of recycled fiber addition on the thermal properties of concrete, such as thermal diffusivity, thermal conductivity and specific heat. Three types of recycled fiber were tested, polypropylene, glass and steel, all fully obtained during the waste recycling process and have not been tested in the available literature so far. Thus, the presented results make an important contribution to this area of research and partly decrease the environmental damage caused by the construction industry.

## 2. Materials

### 2.1. Specimen Preparation

The materials used in this study are Portland cement (CEM I 52.5R), according to EN 197-1:2012 [56], and tap water. As a hardening admixture, a polycarboxylate deflocculant was added. The chemical composition of cement is given in Table 1, and the basic physical–strength properties, measured as per EN 196-6:2019-01 [57] and EN 196-1:2016-07 [58], are given in Table 2. 

**Figure 1:**
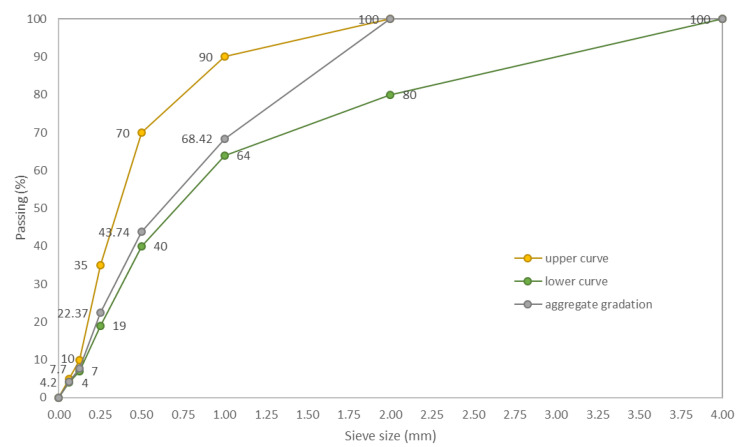
Gradation of crushed granite aggregate.

Crushed granite aggregate with a fraction of 0.063–2.000 mm was used as a filler in all concrete mixtures. Upper and lower sieve curves were determined in accordance with the EN 12620+A1:2010 [60] standard for natural aggregate with a fraction up to 4.0 mm. As presented in Figure 1, the filler is heterogeneous (grain size index C_U_ = 5.72 and C_C_ = 0.96) and well compacted [61].

An increase in the workability of the mixtures was accomplished, with a significantly reduced amount of water, by adding a chemical admixture to fiber-reinforced concrete. A water/cement ratio (w/c) of 0.3 was able to be maintained because of a low-alkaline liquid chemical admixture added. It was based on an aqueous solution of modified polycarboxylic ethers (melamine and silanes/siloxanes) and free of chlorine. The chemical composition of the admixture is presented in Table 3. It is worth mentioning that the additive application limited the phenomenon of “bleeding.” 

### 2.2. Polypropylene Fiber

**Figure 2:**
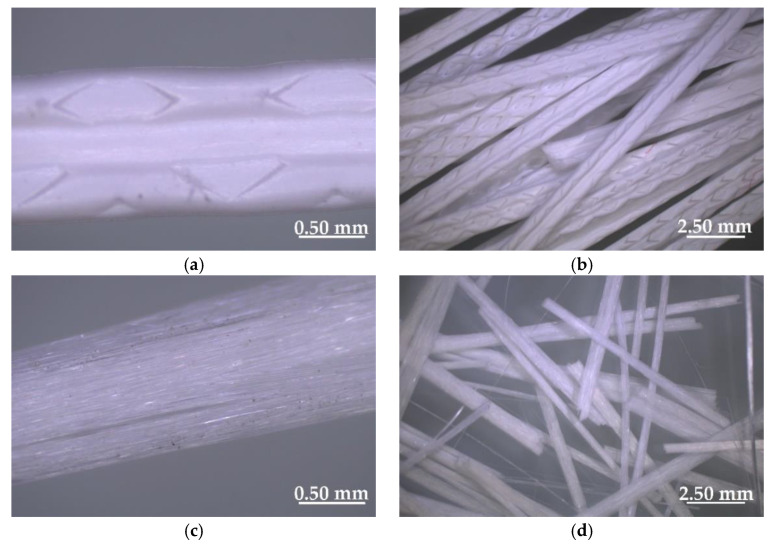

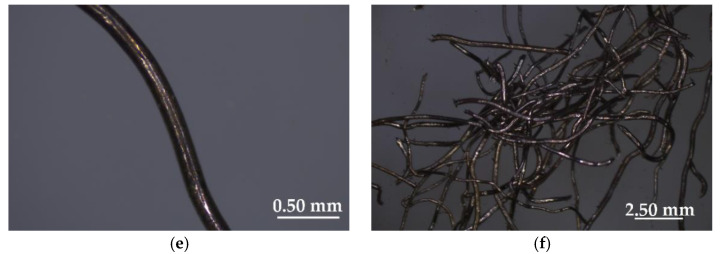
Recycled fibers: (**a**) polypropylene—one fiber; (**b**) polypropylene—a lot of fibers; (**c**) glass—one fiber; (**d**) glass—a lot of fibers; (**e**) steel—one fiber; (**f**) steel—a lot of fibers.

Polypropylene fiber (PF) was made fully from plastic packaging waste and modified by the extruder to increase its adhesion to the cementitious mix (Figure 2a,b). The PF manufacturing process involved thermal and mechanical treatment. Warmed up polypropylene was pressed into the extruder and cut into a regular length on its way out. Fiber of about 31 mm in length (31.1 ± 0.5 mm) and 1.000 microns in diameter was used in this research. The fiber tensile strength was about 520 MPa. Table 4 presents the basic properties of the tested fiber.

### 2.3. Glass Fiber

Glass fiber (GF) obtained from waste materials was used in this study (Figure 2c,d). The fiber was made from the same type of product: chemically identical glass packaging (bottles). The manufacturing process of glass fiber involved the flow of molten glass through electrically heated platinum alloy sleeve plates, and its gravity fell, which dispersed glass into fine fibers. After cooling, glass fibers were coves with sizing and cut to an appropriate shape. The glass fiber was about 49 mm in length (49.3 ± 0.5 mm) with a tensile strength of 1700 MPa. The basic properties of the tested fiber are presented in Table 4.

### 2.4. Steel Fiber

The steel fiber (SF) used in this study was obtained by the process of recycling old tires (Figure 2e,f). After cutting the rubber by machine, steel fibers were separated from the rubber and stored in one place as they are considered byproducts of this process. The final SF had a waved and hooked shape with a diameter of around 0.15 mm and length of about 25 mm (25.2 ± 0.5 mm), and its properties are shown in Table 4. The steel fiber was made from high-carbon steel with a tensile strength of around 2850 MPa. 

### 2.5. Concrete Mixture Composition

Four different types of concrete mixtures (one without fibers and the rest with different contents of fibers) were produced. The fiber content was 0.50, 0.75 and 1.00 wt.% (by mass of the cement), marked in this article as 0.50X, 0.75X or 1.00X, where X is the type of fiber (polypropylene (PF), glass (GF) or steel (SF)). The base recipe (without fiber) was marked as BM. In total, to investigate the effect of fiber content on the thermal properties of the cementitious mix, ten different mixtures were produced (see Table 5). A constant w/c ratio of 0.3 was used for all mixtures. The admixture content was 3.0% of the mass of cement. 

### 2.6. Concrete Mixture Production

All dry components, with fiber addition, were mixed together for 2 min. After that, wet components were added to the concrete mix. The whole mixing process lasted for 5 min. All samples were produced in laboratory conditions (21 °C temperature and 50% humidity) and stored in water according to EN 12390-2:2019-07 [62].

## 3. Methodology

### 3.1. Test on Concrete Mix

#### 3.1.1. Initial and Final Setting Time

The initial and final setting times were measured with the Vicat apparatus (Merazet, Poznań, Poland). Immediately after the mixing process was over, the Vicat mold was well compacted and filled with cement paste. Next, it was placed in the Vicat apparatus, and the plunger was removed to penetrate the paste. The initial time was measured when the penetration of the 1 mm diameter needle was just above 5 mm from the bottom of the mold base. After that, the 1 mm diameter needle was changed to a needle with an enlarged 5 mm hollow cylindrical base. The setting time was noted when this needle made an impression on the surface of the concrete but did not penetrate it.

#### 3.1.2. pH Value

The pH value was determined using a pH meter (Testo, Pruszków, Poland). Tests were carried for a fresh mix with 10 mL of liquid phase as a 10% water solution of mortar according to PN-B-01810:1986 [63]. After the measurement stabilization for three minutes, the pH value was noted. Five samples of each diluted concrete mixture, with and without fiber addition, were investigated. 

#### 3.1.3. Viscosity

The viscosity test was carried out with a DV3T viscometer (AMETEK Brookfield, Middleborough, MA, USA). The maximum speed of 200 RPM and the cycle duration of 20 s were taken as the boundary conditions of the procedure. Each concrete mixture, with and without fiber addition, was measured three times, and the average values were calculated. 

### 3.2. Test on Final Concrete Samples

#### 3.2.1. Porosity

Three cubic samples, with dimensions of 150 × 150 × 150 mm^3^, from each series and each mix were used for testing. The actual dry mass of the samples was weighed and recorded. Then, they were put into a water bath at a temperature of 20 °C and a density of 998 kg/m³. After complete immersion, the samples were left in the water until the weight changes over 24 h were less than 0.2%. After removing the samples, they were wiped with a damp cloth, and the weight of the water-saturated sample was determined. After that, the samples were dried in a laboratory dryer at a temperature of 80 °C, and then the dry weight of the samples was measured. Based on the obtained results and the difference between the weights of dry and wet samples, the porosity was calculated.

#### 3.2.2. Density

The density of hardened concrete samples was investigated using 150 × 150 × 150 mm^3^ standard cubes as per EN 12390-7:2019-08 [64]. Ten specimens were tested for each concrete mix.

#### 3.2.3. Thermal Properties

Thermal conductivity, thermal diffusivity and specific heat were included in the scope of the research. Based on the temperature response analysis to the heat flow impulses of the material, all measurements were made. To do so, an ISOMET2114 analyzer (Applied Precision Ltd., Bratislava, Slovakia) was used. Heat flow was excited by the resistor heater electrical heating inserted into the probe, which was in direct contact with the tested specimen. Probes with a 60 mm diameter and evaluated material with a minimum 25 mm thickness were used. The thermal conductivity evaluation and heat capacity volume were based on temperature records as a function of time taken periodically within an unlimited medium heat propagation. Five measurements in total of each sample were taken at different locations, and the temperature ranged between −15 and 60 °C. Ten cubes (150 × 150 × 150 mm^3^) of each concrete mix were tested this way, and the final result was calculated as the measurements’ average.

## 4. Results and Discussion

### 4.1. Initial and Final Setting Time

**Figure 3:**
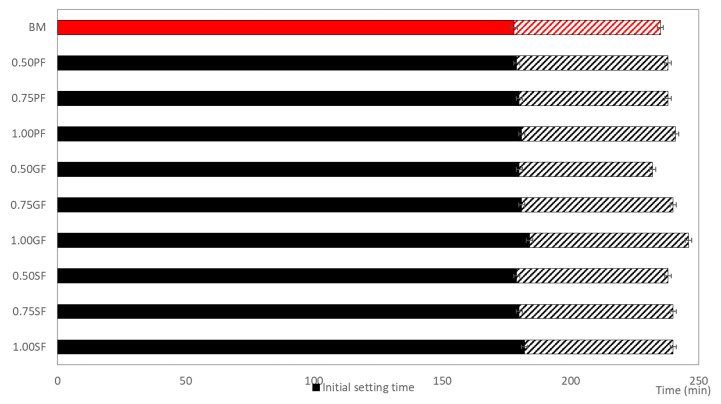
Initial and final setting time distribution of the tested mixtures: samples with fibers marked as black, BM marked as red.

The initial and final setting time distribution for the ten mixtures (one without fibers and the rest with different contents of different fibers) are presented in Figure 3. The initial and final setting time distribution for BM (no fibers) is marked in red, and all modified mixtures (containing fibers) are marked in black. The highest initial and setting times were obtained for 1.00GF, but the occurring difference fits within the error limit of the measurement method. Therefore, it can be concluded that the addition of fiber to the cement mix had no effect on the initial and final setting times. The same conclusion has been made by Małek et al. [29,49], who reported no influence of glass and polypropylene fibers on the initial and final setting time of concrete mortars.

**Figure 4:**
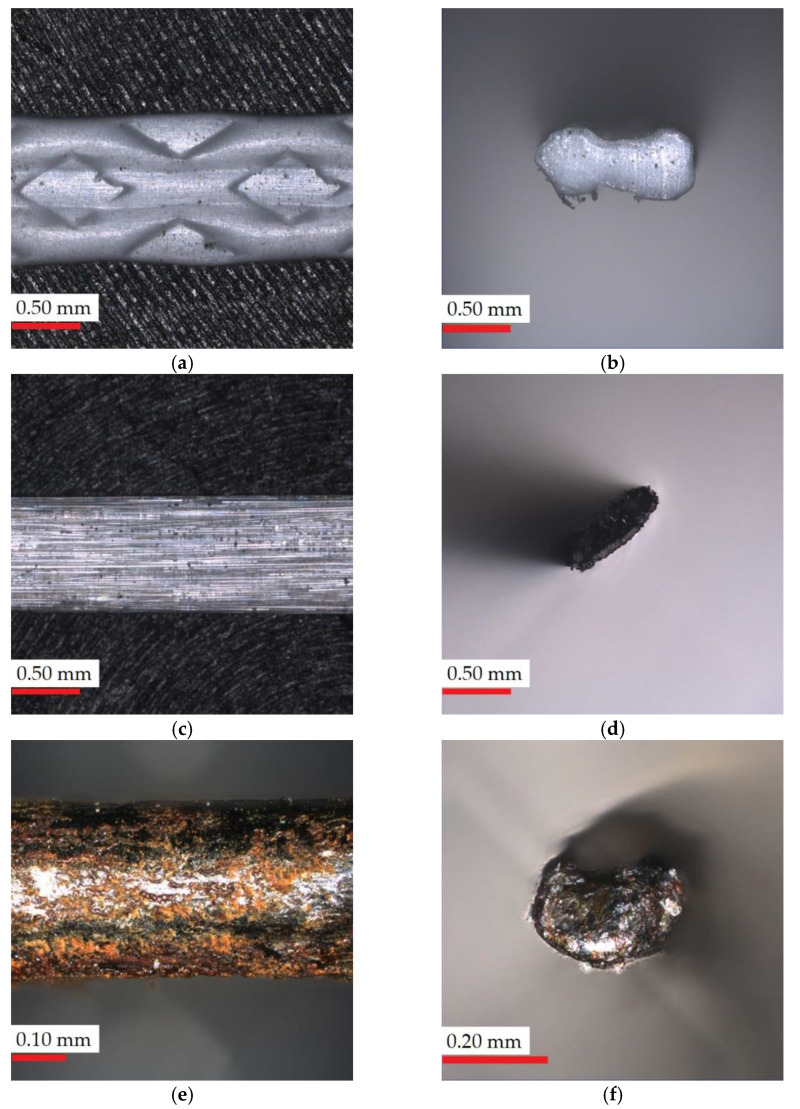
Light microscopy images: (**a**) polypropylene fiber surface; (**b**) polypropylene fiber cross-section; (**c**) glass fiber surface; (**d**) glass fiber cross-section; (**e**) steel fiber surface; (**f**) steel fiber cross-section.

The surfaces of PF, GF and SF are without roughness and smooth (Figure 4), therefore no agglomeration was observed during mixing. Only polypropylene fibers have an additional pattern, but this is created through a special mold, which does not give sharp edges and a rough structure. The fibers were distributed evenly in the mixture (they did not float to the surface or sink to the bottom), which may be related to the use of a chemical admixture. 

### 4.2. The pH Test

The pH values of all recipes exhibit the alkaline reaction (see Table 6). All pH measurements are on the verge of error; therefore, it can be concluded that polypropylene, glass and steel fiber addition did not affect the pH of the concrete mix. However, the concrete mixtures did not reach the melting temperature of any fiber material or even its softening temperature during the concrete production and later care, which could change the pH value. A similar trend was also reported by Małek et al. [29], who tested the pH values of concrete mortars with fiber reinforcement and reported results in the range from 12.83 ± 0.03 to 12.97 ± 0.03.

### 4.3. Viscosity

**Figure 5:**
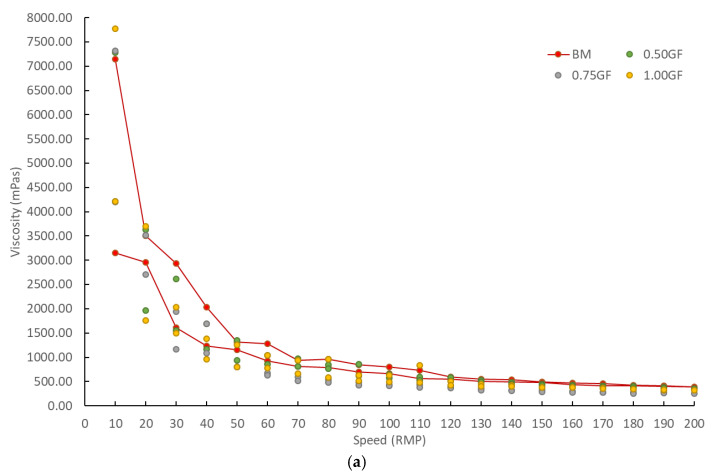

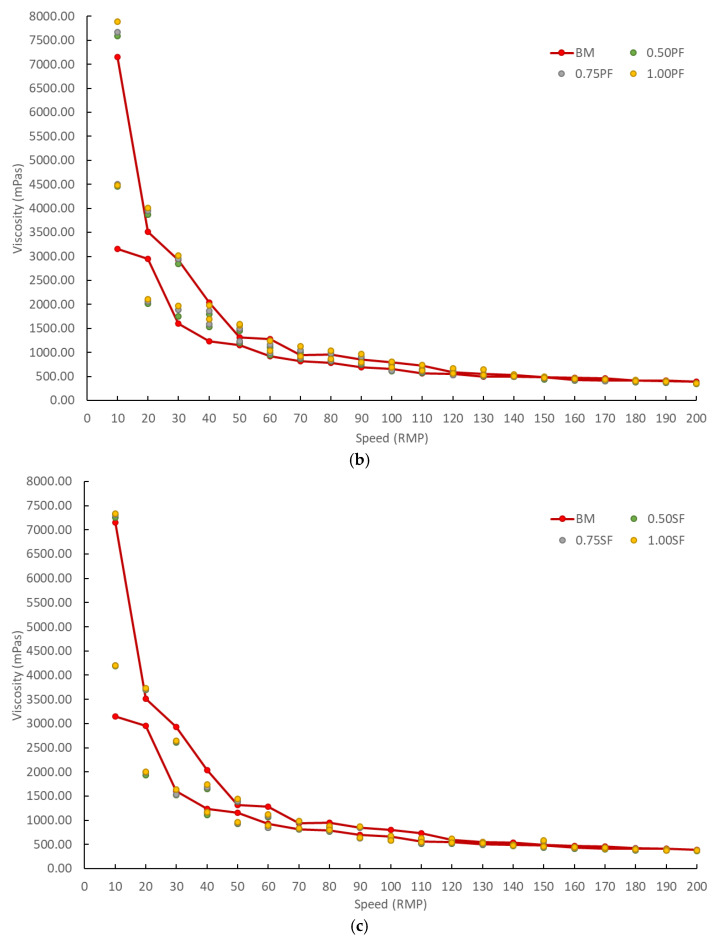
Viscosity test results of the tested mixtures: (**a**) glass fiber concretes; (**b**) polypropylene fiber concretes; (**c**) steel fiber concretes.

The workability of all concrete mixtures is presented in Figure 5. With the addition of polypropylene fibers, the viscosity significantly increased for all speeds. This is the result of intermolecular forces between particles within a fluid, especially between other components and polypropylene fiber, due to its additional pattern (Figure 4a). An increasing trend in viscosity as the content of each type of fiber increases can also be observed. This may affect the workability of mixtures. The same conclusion was reported by Wang et al. [47], who determined that fiber addition higher than 0.5% caused poor workability and the incomplete compaction of concrete. However, in this study, in the mixing process, no such impact was noted. This was probably due to a higher admixture content used compared to Wang’s study [47].

### 4.4. Porosity

Based on the test results (Table 6), it can be observed that the fiber ratio affects the number of pores in the concrete mix. With the increase in the fiber ratio in the concrete composite, the number of pores increased. The highest values of porosity were obtained for 1.00GF samples (7.2%). This is in line with the observation of Söylev et al. [65], who tested concrete mixtures with the addition of polypropylene, glass and steel fiber. For all three types of fiber, a slight increase in the initial increase was observed, and the relationship connection between that and the lower resistivity test results was determined. Their control concrete established a porosity of 2.9% with a 0.45 water/cement ratio. After modification with 0.1% glass and 0.1% polypropylene fiber, its porosity increased to 5.0% and 4.2%, respectively. Furthermore, their tested samples, with the addition of 0.5% steel fiber addition, showed a 3.5% increase in porosity compared to the reference sample. This analogous trend was reported by Ali [45] and Liu [66], which, as they mentioned, it may be a result of the interfacial transition zone formation around fibers.

### 4.5. Density

A slight influence of fiber addition in the density of concrete compared to the base sample can be observed (Table 6). The highest increase in density was observed for samples with steel fibers, 0.7% for 0.50SF, 0.8% for 0.75SF and 0.9% for 1.00SF, compared to BM. The increase in density with an increase in steel fiber content in concrete was also reported by Algourdin et al. [32]. They obtained about a 4.3% increase for the addition of 12.6% steel fiber. Furthermore, the tested glass fiber concrete samples showed an increasing trend in density. The highest increase of 0.3% was determined for 1.00GF samples. Similar results were demonstrated by Madhkhan and Katirai [67], who presented a 0.9% increase in density after adding 1.5% glass fibers to the concrete mixture. Polypropylene fiber concrete showed the lowest increase in density (0.2%, 0.3% and 0.3% for 0.50PF, 0.75GF and 1.00GF samples, respectively). A slight increase in density for the addition of polypropylene fibers was also reported by Altalabani et al. [68]. They demonstrated the highest increase in density equal to 1.16% for samples with 0.57% polypropylene fiber addition, which is comparable to that obtained in this study, or a 0.2% increase. Such a little change in all presented density test results can be caused by the low weight and small size of the fibers, which are able to fit between the aggregate of the mixture. 

### 4.6. Thermal Properties

**Figure 6:**
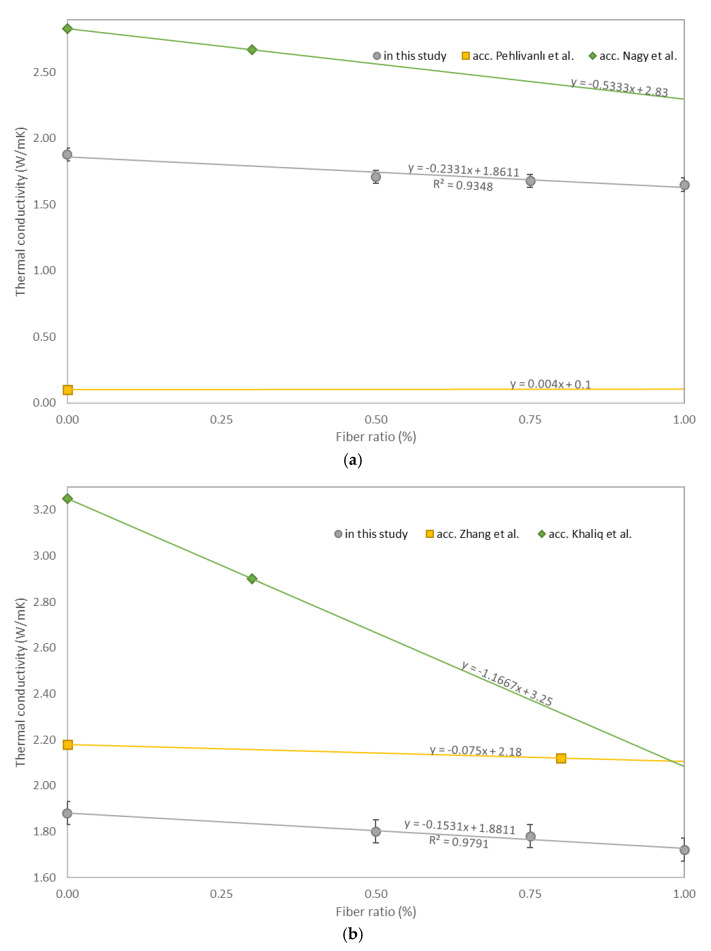

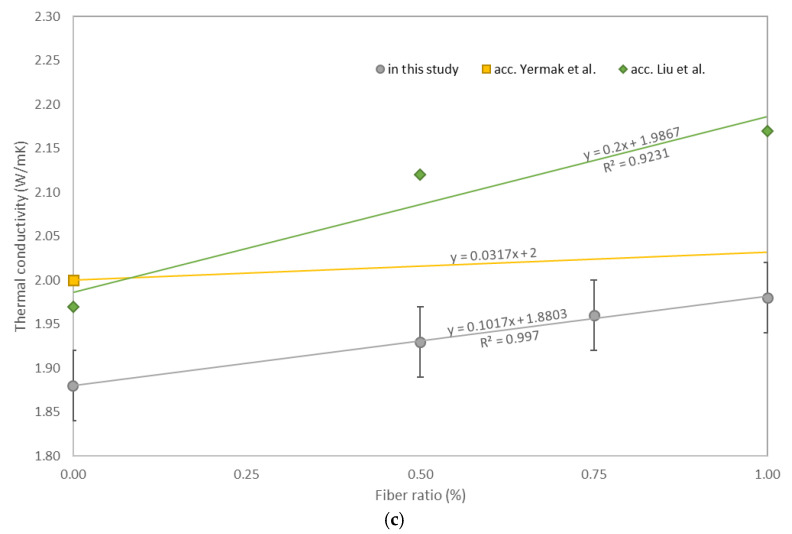
Thermal conductivity test results: (**a**) glass fiber concretes; (**b**) polypropylene fiber concretes; (**c**) steel fiber concretes.

The thermal conductivities of the tested samples with different fiber types are shown in Figure 6a–c. As the fiber content increased, the average thermal conductivity decreased for polypropylene and glass fiber concretes. An opposite trend can be noted for steel fiber concretes. It can be observed that, with fiber content, thermal conductivity increased compared to plain concrete (BM). The obtained correlation was linear; moreover, the coefficients of determination were above 0.9, which means a good fit. As Table 6 stands, the lowest values of thermal conductivities were obtained for 1.00GF samples, which, compared to BM, show a 12% decrease in value. For 0.50GF and 0.75GF samples, a decrease in thermal conductivity was noted at 9% and 10%, respectively. This phenomenon is closely related to the changes in porosity. Nagy et al. [69] also reported lower values in thermal conductivities for glass fiber concrete. With the 0.3% addition of glass fiber concrete, they achieved a decrease in thermal conductivity from 2.83 to 2.67 W/mK, which is about 5.6%. An opposite trend was reported by Pehlivanlı et al. [52], who tested thermal conductivities of glass fiber concretes. Samples modified with 2.50% glass fiber (density 2.54–2.60 g/cm^3^) showed about a 3.3% and 10.0% thermal conductivity increase depending on the plain concrete recipe. This, however, was indicated by the type of concrete that they tested (autoclaved aerated concrete), which had very low thermal conductivity, in the range of 0.11–0.13 W/mK, and high porosity. As the fibers took air void places, thermal conductivity rose, which can be expected in foam or aerated concrete. The tested polypropylene fiber concretes showed a decrease in thermal conductivity as well. For fiber additions of 0.50%, 0.75% and 1.00%, values lowered by 4.3%, 5.3% and 8.5%, respectively, compared to BM. The lowest thermal conductivity was reported for 1.00PF, which was equal to 1.72 W/mK. Zhang et al. [50] also tested the thermal properties of polypropylene fiber concrete. They used polypropylene fibers, 6 mm in length, 17 µm in diameter and with the same 0.3 water/cement ratio, and obtained a 2.8% decrease in thermal conductivity after adding 0.8% fiber to plain concrete. Khaliq et al. [44] reported this trend as well; however, they obtained a much higher decrease in thermal conductivity after 0.3% polypropylene fiber addition (about 13.8% decrease). This may be the result of different fiber dimensions, as Khaliq et al. used polypropylene fibers 20 mm in length. Longer fibers fill more of the sample matrix compared to shorter fibers and create larger uniform spots with different thermal properties, which contributes to a change in thermal conductivity. Therefore, it can be seen that the thermal properties depend not only on the type and number of fibers but also on their dimensions. Steel fiber concrete, on the other hand, showed an increase in thermal conductivity, respectively, by 2.7%, 4.3% and 5.3% for 0.50SF, 0.75SF and 1.00SF samples. A correlation between steel fiber addition and an increase in thermal conductivity was reported by Yermak et al. [46] as well. They presented an increase in thermal conductivity from 2.0 up to 2.2 W/mK after the 60 kg/m^3^ addition of steel fibers (an increase by 10%). Liu et al. [53] studied this topic also and arrived at a similar conclusion. In their work, a 7.6% and 10.2% increase in thermal conductivity compared to plain concrete was noted for samples containing 0.50% and 1.00% steel fibers, respectively, with a constant w/c ratio equal to 0.4.

**Figure 7:**
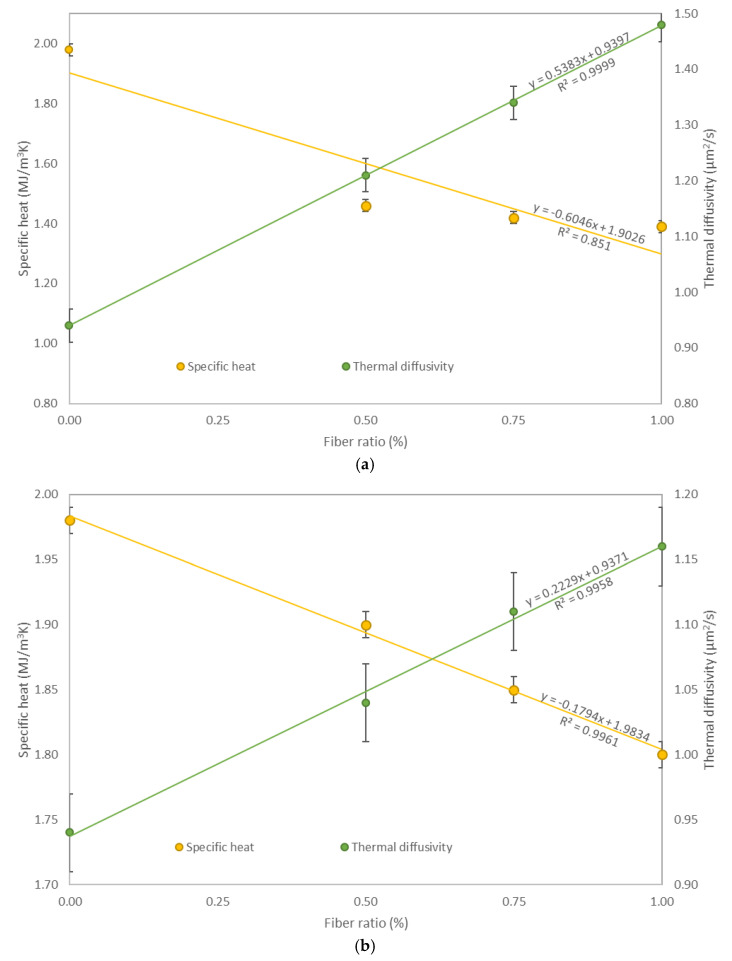

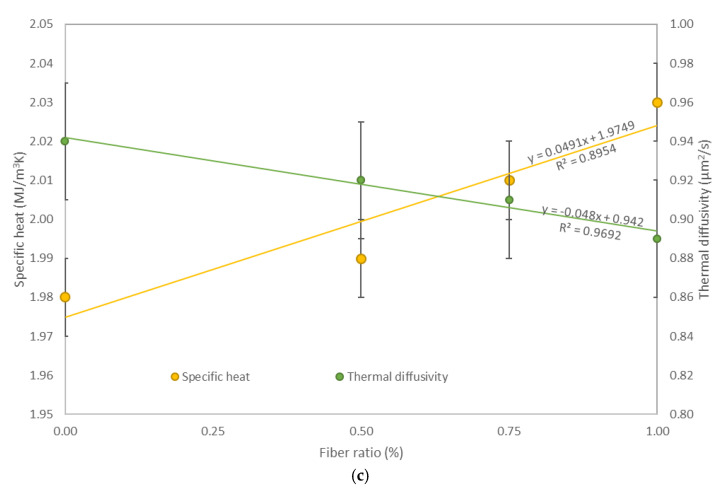
Specific heat and thermal diffusivity test results: (**a**) glass fiber concretes; (**b**) polypropylene fiber concretes; (**c**) steel fiber concretes.

The specific heat measured in this study, presented in Table 6, should be regarded as a fundamental specific heat of whole fiber-reinforced composites. Tested samples modified with glass fibers showed a decrease in specific heat compared to the reference sample (BM). The lowest specific heat was reported for 1.00GF (1.39 MJ/m^3^K), and its value was about 30% lower than plain concrete, see Figure 7. Other modifications made with glass fibers also showed a high effect on specific heat. The specific heat decreased by about 26% and 28% for 0.50GF and 0.75GF samples, respectively. The influence of polypropylene fiber on specific heat was also studied, and the highest number of fibers indicated the highest decrease in specific heat. For 0.50PF, 0.75PF and 1.00PF samples, the obtained values of 1.90 MJ/m^3^K, 1.85 MJ/m^3^K and 1.80 MJ/m^3^K were lower by about 4%, 7% and 9% compared to BM, respectively. Steel fiber concrete, however, did not continue the decreasing specific heat trend presented above, and opposite phenomena were observed. Compared to plain concrete, samples containing steel fibers showed an increase in specific heat. The highest increase was reported for the highest number of fibers (about 2.5%). The same trend was reported by Algourdin et al. [32], who presented about a 30% increase in specific heat for steel fiber concrete compared to plain concrete. Such a difference in results, compared to this study, may be the result of the high dosage of fibers used by Algourdin et al. [32], which was equal to 60kg/m^3^, an equivalent of 12.6% cement mass used in their mixtures.

The thermal diffusivities of the tested samples are presented in Figure 7. The thermal diffusivity for 1.00GF samples was equal to 1.48 µm^2^/s (about 57% higher than plain concrete). Due to an increase in this property by 42.5% and 28.7% for 0.50GF and 0.75GF samples, respectively, it can be concluded that the higher the glass fiber content, the higher the thermal diffusivity of concrete. This trend applies also to concrete reinforced with polypropylene fibers. As this study shows, samples with a 0.50%, 0.75% and 1.00% addition of polypropylene fibers obtained a thermal diffusivity of 1.04 s, 1.11 and 1.16 µm^2^/s, respectively. It is worth mentioning that the highest increase in this modification was equal to 23.4%. Opposite phenomena were reported for steel fiber concretes as their thermal diffusivity decreased with increasing fiber content. The lowest values of thermal diffusivity (0.89 µm^2^/s) were noted for 1.00SF samples, which was about 5.3% less compared to BM. Fu and Chung [70] also reported a decrease in the thermal diffusivity of fiber-reinforced concrete. They obtained 10.8% and 29.7% lower values of thermal diffusivity compared to plain concrete with a 0.50% and 1.00% addition of carbon fibers, respectively. This discrepancy in results may be a result of an additional 0.4% methylcellulose in concrete mixtures and different types of fiber used.

## 5. Conclusions

In the present work, we aimed to clarify the effect of three different types of fibers (glass, polypropylene and steel fibers) on the thermal conductivity, specific heat and thermal diffusivity of fiber-reinforced concretes. As there was no agglomeration process observed during mixing, it is possible to utilize recycled waste in fiber form by adding it to concrete mixtures. The tested materials can be applied in a precast concrete wall, as they require high thermal properties. On the basis of this study, it has been proven that the thermal properties of fiber-reinforced concrete depend on the type of fiber used in the manufacturing process. The obtained results of the fiber-reinforced concretes, through our experimental research, are as follows:

- No significant influence on the initial and final setting time, pH values and density of concretes was determined after the addition of polypropylene, glass and steel fibers;
- The workability of all concrete mixtures was not affected by fiber addition, which was confirmed by conducted viscosity tests;
- All tested samples showed increased values in porosity compared to the base mixture. The highest porosity was reported for glass fiber concretes: 5.9%, 6.7% and 7.2% for 0.50GF, 0.75GF and 1.00GF samples, respectively. For concrete with 1.00% glass fiber, the obtained porosity was over three times higher compared to the reference sample;
- Thermal conductivity tests yielded values from 1.65 to 1.98 W/mK. The lowest thermal conductivity was reported for 1.00GF, which showed a decrease in values by 12% compared to BM. The highest thermal conductivity was reported for 1.00SF samples, as their results were 5.3% higher than BM;
- A decreasing specific heat trend with increasing polypropylene and glass fiber dosage was reported. An opposite trend was defined for concretes reinforced with steel fibers. Specific heat values were in the range of 1.39–2.03 MJ/m^3^K;
- 1.00SF samples showed the lowest thermal diffusivity (0.89 µm^2^/s), and 1.00GF samples showed the highest thermal diffusivity (1.48 µm^2^/s). For polypropylene and glass fiber concrete, an increase in thermal diffusivity was observed with increasing fiber amount. Steel fiber concrete showed a contrary trend, as the values were much lower compared to BM.

This research is part of a wider study of sustainable solutions that can be introduced to the building sector and contribute to reducing environmental damage caused by the construction industry. The large-scale deployment of waste materials as mixed components could make the building sector more environmentally friendly and target the Paris Agreement levels.

## Figures and Tables

**Table 1:** Cement chemical composition [59].

Compositions	SiO_2_	Al_2_O_3_	Fe_2_O_3_	CaO	MgO	SO_3_	Na_2_O	K_2_O	Cl
Unit (vol. %)	19.5	4.9	2.9	63.3	1.3	2.8	0.1	0.9	0.05

**Table 2:** Cement physical and strength properties [59].

Specific Surface Area (m^2^/kg)	Initial Setting Time (min)	Compressive Strength After (MPa)	
		2 days	28 days
512.7	163	38.0	70.8

**Table 3:** Chemical composition of admixture.

Compositions	O	Na	Si	K
Unit (vol. %)	77.7	14.9	4.8	2.6

**Table 4:** Properties of fibers.

Type of Fiber	Average Thickness (μm)	Average Circumference (μm)	Length (mm)	Tensile Strength (MPa)
PF	1155.0 ± 10.0	538.5 ± 0.5	30.4–32.0	520
GF	840.0 ± 10.0	412.1 ± 0.5	48.9–50.4	1700
SF	345.0 ± 10.0	155.6 ± 0.5	24.3–26.1	2850

**Table 5:** Mix proportions (1 m^3^).

Mix Symbol	Cement (kg)	Aggregate (kg)	Water (kg)	Admixture (kg)	Type of Fiber	Fiber Content (kg)
BM	450	1600	135	13.5	–	0.00
0.50PF					PF	2.25
0.75PF						4.50
1.00PF						6.75
0.50GF					GF	2.25
0.75GF						4.50
1.00GF						6.75
0.50SF					SF	2.25
0.75SF						4.50
1.00SF						6.75

**Table 6:** Experimental results of material and thermal properties (average values).

Mix Symbol	Porosity (%)	pH	Density (kg/m^3^)	Thermal Conductivity (W/mK)	Thermal Diffusivity (µm^2^/s)	Specific Heat (MJ/m^3^K)
BM	2.3 ± 0.1	12.81 ± 0.03	2210 ± 2	1.88 ± 0.06	0.94 ± 0.03	1.98 ± 0.01
0.50PF	5.7 ± 0.1	12.85 ± 0.03	2214 ± 2	1.80 ± 0.05	1.04 ± 0.04	1.90 ± 0.01
0.75PF	6.1 ± 0.1	12.87 ± 0.04	2216 ± 3	1.78 ± 0.06	1.11 ± 0.03	1.85 ± 0.01
1.00PF	6.4 ± 0.1	12.89 ± 0.03	2217 ± 2	1.72 ± 0.05	1.16 ± 0.03	1.80 ± 0.01
0.50GF	5.9 ± 0.1	12.83 ± 0.03	2215 ± 2	1.71 ± 0.05	1.21 ± 0.04	1.46 ± 0.01
0.75GF	6.7 ± 0.1	12.89 ± 0.03	2217 ± 3	1.68 ± 0.06	1.34 ± 0.03	1.42 ± 0.01
1.00GF	7.2 ± 0.1	12.97 ± 0.04	2219 ± 2	1.65 ± 0.05	1.48 ± 0.03	1.39 ± 0.01
0.50SF	3.2 ± 0.1	12.86 ± 0.03	2226 ± 2	1.93 ± 0.05	0.92 ± 0.04	1.99 ± 0.01
0.75SF	3.7 ± 0.1	12.90 ± 0.04	2227 ± 3	1.96 ± 0.06	0.91 ± 0.03	2.01 ± 0.01
1.00SF	4.0 ± 0.1	12.92 ± 0.04	2230 ± 2	1.98 ± 0.05	0.89 ± 0.03	2.03 ± 0.01

## Data Availability

Data are contained within the article.
